# Quantitative composite testing model based on measurement uncertainty and its application for the detection of phthalate esters

**DOI:** 10.3389/fchem.2023.1191669

**Published:** 2023-09-18

**Authors:** Lina Huang, Yi Hu, Guanwei Li, Caiding Ouyang, Lezhou Yi, Shanshan Wu, Zhenhai Zhu, Tongmei Ma

**Affiliations:** ^1^ Quality and Standards Academy, Shenzhen Technology University, Shenzhen, China; ^2^ Institute for Advanced Study, Shenzhen University, Shenzhen, China; ^3^ School of Chemistry and Chemical Engineering, South China University of Technology, Guangzhou, China; ^4^ Technology Center of Guangzhou Customs District, Guangzhou, China

**Keywords:** composite testing, phthalates (PAEs), quantitative composite testing model, measurement uncertainty, toys

## Abstract

To improve the quantitative detection efficiency of chemical analysis and reduce the detection cost, the sample pass rate was estimated and mathematical statistics were used to calculate the optimal group size (*K*_opt_) of the composite testing to save on the maximum workload. A quantitative composite testing model was developed based on chemical analysis measurement uncertainty. Using this model, the maximum allowable number of composited samples (*K*_max_) is first calculated using parameters of regulated limits (*L*), limit of quantification (*LOQ*), and method measured uncertainty (*U*_*rel*_) to ensure that the sensitivity of the composite testing can meet the limit requirements. Finally, the appropriate composite group size (*K*_a_) can be obtained by creating a balance between *K*_opt_, *K*_max_, and the practical information used for that particular test. Furthermore, based on a constructed model, a practical quantitative composite testing method of 3–10 samples was established for the routine detection of toy phthalates (PAEs). The experimental results showed that the quantitative limits of 7 PAEs were 9.1–41.8 mg/kg, the relative expansion uncertainties were 16.6%–23.2%, and the recovery rates were 91.0%–112.3%, with a relative deviation of less than 10%. All these meet international PAEs standards. Compared with the traditional individual and qualitative composite testing, this model will not decrease the detection sensitivity, but can save up to 17.9%–80.4% of the workload when it is employed in toy PAEs testing with the pass rate of 80%–99%. This quantitative composite testing method will be implemented in the coming revision of ISO 8124-6 toy PAEs standards.

## 1 Introduction

Composite testing is a method in which multiple samples are mixed and a single test is performed, while the results can determine whether unqualified or defective samples are included in the group. If the composite testing results are judged to pass, all samples in the group are qualified. On the contrary, the test is failed, all the samples in the group are individually tested to identify the unqualified samples. The composite testing method was first proposed by Dorfman in 1943 and was applied for the diagnosis of syphilis in soldiers (Dorfman, 1943). However, the composite of multiple samples leads to a decrease in detection sensitivity, which seriously affects the detection accuracy. Thus, the limitation of instrument sensitivity at that time prevented the further popularization of this method. Along with the development of science and technology, the sensitivity of various detection instruments have improved significantly, resulting in the wide application of composite testing in the medical field for the rapid screening and detection of infectious diseases, such as HIV (Dodd et al., 2002), hepatitis B/C (Offergeld et al., 2005) and the recent outbreak of the novel coronavirus, COVID-19 (Hogan et al., 2020; Nalbantoglu, 2020). At the same time, due to its advantages of high efficiency and low cost, composite testing has gradually become a screening detection method in many fields. For example, industrial production (Du and Hwang, 2006), genetic testing (Kaseniit et al., 2016) and informatics (Kuhn et al., 2008) have brought huge economic and social benefits. An intensive literature survey has revealed that quantitative detection requires higher detection sensitivity than qualitative detection, and the application of the above-mentioned composite testing method is mostly limited to qualitative screening detection. As a result, quantitative detection has been rarely reported on.

Qualitative composite testing aims to analyze whether there is a target substance in the sample group. However, in a quantitative composite testing, it is necessary to quantitatively analyze whether the content of the target substance in a sample group exceeds the limit or regulatory requirements (Sobel and Groll, 1959). For example, *K* samples are weighed and mixed for quantitative testing, and it is assumed that the detected target substance is completely from the minimum mass sample in the group in order to calculate the maximum possible concentration of the target substance. Thus, if it is lower than the limit requirement, the target substance concentrations of all group samples must be lower than the limit, which means that all the samples in the group are qualified. Otherwise, all the samples need to be individually tested for confirmation. When the detection sensitivity is satisfied and the sample qualification rate is high enough, quantitative composite testing can determine whether the sample is qualified using fewer tests, which has been proven to be more efficient and economical for rapid screening and detection under most circumstances, compared with the qualitative composite testing.

A large number of literature provide information on in-depth research conducted on qualitative composite testing and qualitative composite models from different perspectives have been developed based on the classical composite testing model proposed by Dorfman. However, to the best of our knowledge, there are very few studies that have been conducted on the quantitative composite detection model and only a small number of standards are related to quantitative composite testing. For example, both the International Standard Organization (ISO) 8124-6 “Safety of Toys 2018-Part 6: Certain Phthalate Esters in Toys and Children’s Products” (ISO, 2018) and Chinese standard GB 22048-2015 “Detection of Phthalate plasticizers in Toys and Children’s Products” (GBT, 2015) have provided quantitative composite testing methods for the determination of phthalate esters (PAEs) in toy materials. Nevertheless, the relevant parameters and applicable scope of the method for the above standards are still deficient: no more than 3 samples are grouped for a quantitative composite test and the largest number of mixed samples is limited to 3 in a group and cannot take full advantage of the composite testing method. Additionally, the selection of the group size is based on conservative empirical estimation rather than scientific theoretical calculations and relevant experimental demonstration, in which, empirical safety factors, instead of the measurement uncertainty that is commonly used in the testing industry, are employed. Therefore, the accuracy of the measurement results cannot be guaranteed.

In this study, for the first time, we constructed a mathematical model of quantitative composite testing based on the measurement uncertainty. This model aimed to solve the existing problems with quantitative composite testing. For instance, it is ambiguous in scope, limited in detection efficiency, and it can be difficult to guarantee the accuracy of detection results. According to parameters, such as the regulatory limit, method quantification limit, and measurement uncertainty, the value range of the group size *K* allowed by the detection sensitivity can be calculated, which ensures the accuracy of the quantitative results. The final appropriate number of samples *K*_a_ in a group can be determined using mathematical statistics. Using this model, the quantitative results of composite testing can also be obtained and assessed. Since the detection of PAEs in toys or children’s products has the characteristics of a high sample qualification rate and higher regulatory limit than the quantification limit of the method, it is suitable to use the method of quantitative composite testing (Staples et al., 1997; Moore, 2000; Matsumoto et al., 2008; Wang et al., 2015), and this constructed model was then applied for the detection of PAEs in toy materials for verification. Experiments showed that when the detection sensitivity was satisfied and the sample qualification rate was high enough (for example, 95%), the quantitative composite testing method could greatly improve the detection efficiency and reduce testing costs.

## 2 Construction of the quantitative composite testing model

The key to conducting accurate quantitative composite testing is to select an appropriate group size (*K*_a_), which represents the number of sub-samples within a testing group. These sub-samples are combined and tested at once, and the final selected appropriate *K*_a_ can enhance the detection efficiency and ensure the accuracy of the test results.

To achieve accurate quantitative composite testing, the mathematical model developed in this study is based on the measurement uncertainty commonly used in the detection field to obtain the confidence interval of the measured values. The measurement uncertainty, which characterizes the dispersion of the measured values, plays a crucial role in assessing the measurement quality and ensuring the accuracy of the results. The measurement uncertainty assessment prescribed in the international standard ISO/IEC (International Electrotechnical Commission) 17,025 (ISO, 2017) has been widely adopted by testing laboratories worldwide. It is employed in the calibration of laboratory testing methods and instruments, ability verification, conformity assessment, and actual testing processes, taking into account factors such as personnel, methods, equipment, and environmental influences that may affect detection results. In this work, based on the classical algorithm of optimal group size proposed by Dorfman (Dorfman, 1943), and to consider the limitation of instrument detection sensitivity on group size, a mathematical model has been constructed. This model is used to determine the maximum allowable group size *K*_max_, the optimal group size *K*_opt_, and the appropriate composite group size *K*_*a*_. Furthermore, the model enables the calculation and assessment of quantitative composite testing results.

### 2.1 The optimal group size *K*_*opt*_

The optimal group size *K*_opt_ can be used to identify all the unqualified samples by the lowest detection times. However, when the group size is too large, the probability of unqualified sub-samples in a composite sample group that needs to be tested individually will increase. This reduces the detection efficiency, making it essential to select the most appropriate group size based on the estimated sample qualification rate. According to the classical algorithm of the optimal group size proposed by Dorfman in 1943 (Dorfman, 1943), the total number of samples is *n*, and *K* represents the number of mixed samples in a group. There are *n/K* groups in total. Assuming that the qualification rate is *q*, and the false-positive probability is *Z*, then according to the probability theory, the probability that all *K* samples are qualified is *q* ^*K*^ *-Z*, and one sample group only needs to be tested once to determine that all *K* sub-samples are qualified. Additionally, the probability of unqualified sub-samples in the *K* sample group is 1-*q* ^*K*^ *+ Z*. To identify unqualified samples, the sub-samples in the group should be tested individually. Consequently, this group needs to be tested 1 + *K* times in total.

As PAEs are intentional additives in high concentrations (>1%), the targeted PAEs with detectable concentrations in a batch are rare, meaning that *Z* is far smaller than *q* ^*K*^. Therefore, the probability of *Z* can be ignored. Formula (1) can be derived and used to calculate the expected total average detection times *N* with different qualification rate *q* and different group size *K*.

$$N=\left(K+1\right)\times \left(1-q^{K}\right)\times \frac{n}{K}+q^{K}\times \frac{n}{K}=n\times \left(1-q^{K}+\frac{1}{K}\right)$$ (1)

When N < n, the saved testing workload (*S*) is given by Formula (2).

$$S=1-\frac{N}{n}=1-\frac{n\left(1-q^{K}+\frac{1}{K}\right)}{n}=q^{K}-\frac{1}{K}$$ (2)

If the result is not detected or the qualification rate *q* is fixed, when the saved testing workload *S* reaches the maximum, the composite group *K* is the optimal group size in theory.

Table 1 lists the testing time of a single sample obtained from Formula (1), with different qualification rates of samples (*q*) and group sizes (*K*), on the premise that the sensitivity of the detection system is sufficient. When *q* is 70% and *K* is 3, the testing time is 0.99, then *S* is 0.01, and each sample is expected to save 1% of detection times by comparing with the routine measurement. When *q* is 99% and *K* is 11, the testing time is 0.196, then *S* is 0.804, which can save 80.4% of detection times. From this table we conclude that as the qualification rate *q* becomes higher, this quantitative composite testing method can increasingly reduce the detection workload and save the testing cost.

**TABLE 1:** The expected detection times of a single sample in the group with different qualification rates (*q*) and group sample sizes (*K*).

Group size (*K*)	Qualified rate (*q*, in %)^a^					
	0.69	0.70	0.80	0.90	0.95	0.99
2	1.024	1.010	0.860	0.690	0.598	0.520
3	1.005^[a]^	0.990	0.821	0.604	0.476	0.363
4	1.023	1.010	0.840	0.594	0.435	0.289
5	1.044	1.032	0.872	0.610	0.426	0.249
6	1.059	1.049	0.905	0.635	0.432	0.225
7	1.068	1.061	0.933	0.665	0.445	0.211
8	1.074	1.067	0.957	0.695	0.462	0.202
9	1.076	1.071	0.977	0.724	0.481	0.198
10	1.076	1.072	0.993	0.751	0.501	0.196
11	1.074	1.071	1.005	0.777	0.522	0.196
12	1.072	1.069	1.015	0.801	0.543	0.197
13	1.069	1.067	1.022	0.823	0.564	0.199
14	1.066	1.065	1.027	0.843	0.584	0.203
15	1.063	1.062	1.031	0.861	0.603	0.207

a The value with underline is the minimum testing time of a specified qualification rate *q*.

**FIGURE 1:**
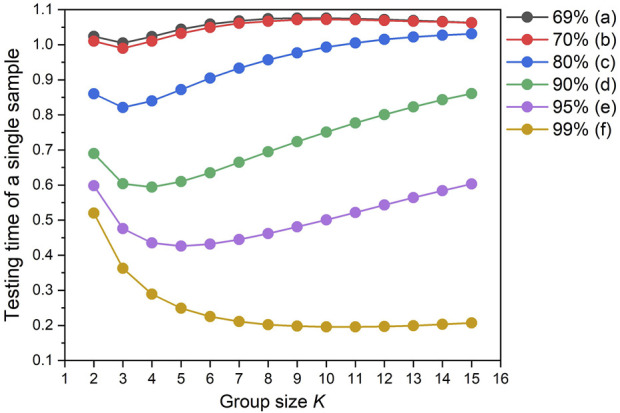
Dependence of the expected detection time of a single sample as a function of the group size (K) and qualification rate (q).

Figure 1 shows the variation trend of expected testing times under different conditions, and the saved testing workload *S* changes in an opposite tendency. For different qualification rates *q*, *S* increases first and then decreases with the increase of the group size *K*, indicating that too many samples in a group will lead to lower detection efficiency.

To reduce detection times, it is necessary to determine the optimal group size *K*_opt_ to obtain the maximum saved testing workload in the quantitative composite testing. As shown in Figure 1A, when *q* is 69%, the expected testing time is more than 1, which means that *S* is less than 0. Hence, it can be concluded that if the sample qualification rate *q* is lower than 70%, composite testing of any group size *K* will lead to more detection times and thus composite testing is not applicable. With the improvement of the sample qualification rate *q*, saved testing workload *S* under the same group size *K* exponentially increase. Normally, comparing with the traditional individual testing, the higher is the *q*, the more detection times can be saved by this quantitative composite testing method. For example, Figure 1F shows that when *q* is 99%, the value of *S* approximately reaches 0.8, saving 80% of the detection times. Therefore, the quantitative composite testing method is recommended when the sample qualification rate *q* is higher than 70%.

### 2.2 The Maximum Allowable Group Size *K*_max_

It is important to consider factors that affect the accuracy of results when determining the composite group size, *K*, rather than relying solely on a mathematical analysis to maximize the saved testing workload. A comprehensive analysis should be conducted to determine the maximum allowable group size (*K*_max_) that guarantees the accuracy of composite testing results. To prevent the occurrence of undetected mixed samples that contain unqualified sub-samples, the group size should be selected within the range of 2 to *K*_max_.

*K*_max_ is based on a comprehensive analysis of factors that affect the precision of the composite testing results. These factors include limits (*L*), the number of concerned substances in the limit (*I*), limit of quantification (*LOQ*), instrument detection limit (*IDL*), and other factors that vary among laboratories due to differences in testing capabilities and material diversity. Therefore, a safety factor (*F*) based on experience and historical data is recommended. Taking these factors into account, Formula (3) has been derived to calculate the maximum allowable group size *K*_max_.

$$K_{\max}=\frac{L\times \left(1-U_{rel\;\max}\right)}{Q_{M,\max}\times I}\times F$$ (3)

$$Q_{M,\max}=\frac{Q_{I,\max}\times V}{m_{\min}}$$ (4)

In Formula (3), *L* represents the maximum regulated limit for the target substance. *U*_*rel max*_ (%) is the maximum value of relative expanded uncertainty among all *U*_*rel*_ values of the tested substance(s). *F* denotes the safety factor of regulated limits. *L*, *I* and *F* are determined by the practical application. *Q*_*M,max*_ is the maximum value of *LOQ* among all *LOQs* of the tested substance(s) for the method. It is the most critical factor, and can be estimated from Formula (4). *Q*_*I,max*_ is the maximum *IDL* value among all *IDLs* of the tested substance(s) for the instrument. *V* represents the final volume of the composite test solution, and *m*_min_ is the minimum mass of test portions in the composite test. *I* denotes the number of substances corresponding to the limit. For instance, the European REACH Directive (European Agency for Safety and Health at Work, 2006) stipulates that the sum concentration of three test items, DINP, DNOP, and DIDP, must not exceed 0.1%. In this case, *I* = 3. If information about the limit, *LOQ*, measurement uncertainty, etc., is available, *K*_max_ can be calculated using Formula (3). The final calculated value of *K*_max_ should be rounded down.

**FIGURE 2:**
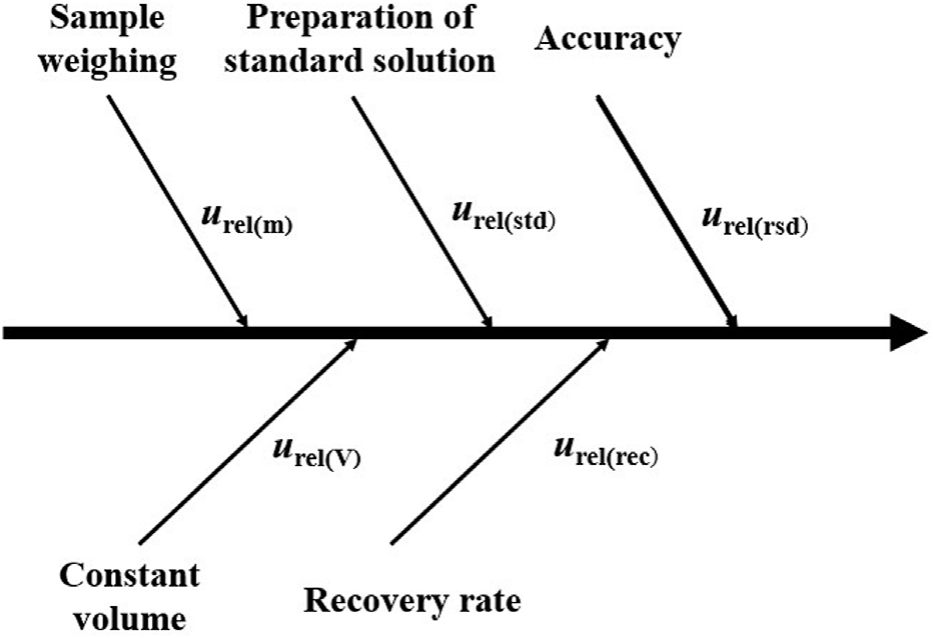
Possible sources of measurement uncertainties.

Before performing quantitative composite testing, LOQ and *U*_*rel*_ in Formula (3) need to be evaluated. *LOQ* refers to the minimum amount of the analyte in the sample that can be quantitatively determined with defined precision and accuracy under the specified experimental conditions. *LOQ* is used to measure detection sensitivity and is generally equal to 10 times the standard deviation of multiple parallel testing results. *U*_*rel*_ is the relative expanded uncertainty close to the regulation limit and is used to indicate the possible dispersion of the measured values (Miekisch et al., 2008). To ensure the validity and accuracy of composite testing results, evaluating the relative expanded uncertainty *U*_*rel*_ is necessary. Currently, for detecting PAEs, most laboratories can work out their own data and use them in the composite testing. Figure 2 shows possible sources of measurement uncertainties.

The relative combined standard measurement uncertainty *u*_*rel*_ mainly consists of the relative standard uncertainty of mass *u*_*rel(m)*_, volume *u*_*rel(V)*_, standard working solution *u*_*rel(std)*_, recovery *u*_*rel(rec)*_, and accuracy *u*_*rel(rsd)*_. The value of *u*_*rel*_ can be obtained from Formula (5), which refers to ISO/IEC GUIDE 98-3-2008 (ISO, 2008).

$$u_{rel}=\sqrt{u_{rel\left(m\right)}^{2}+u_{rel\left(V\right)}^{2}+u_{rel\left(std\right)}^{2}+u_{rel\left(rec\right)}^{2}+u_{rel\left(rsd\right)}^{2}}$$ (5)

Assuming that the testing result conforms to the normal distribution, then the relative expanded uncertainty *U*_rel_ can be calculated using Formula (6), the coverage factor, *k,* mostly equals 2 when the confidence degree is 95%.

$$U_{rel}=k\times u_{rel}$$ (6)

*K*_max_ is calculated according to Formula (3), Formula (4), Formula (5), and Formula (6).

When the regulation or law requires that the combined concentration of multiple target substances lower than the limit, they need to be simultaneously detected. In this case, *K*_max_ is calculated using the maximum values of *U*_rel_ and *LOQ* among the target substances. When *K*_max_ ≤ 1, the quantitative composite testing method is not applicable.

### 2.3 Determination of the final group size *K*_*a*_

The appropriate composite group size *K*_a_ can be affected by various factors, such as regulation limits, *LOQ*, measurement uncertainty, and qualification rate. The laboratory should consider all factors to select a suitable *K*_*a*_. By comparing *K*_max_ and *K*_*opt*_, *K*_a_ should be the smallest value of *them*. However, considering practical constraints, *K*_*a*_ is limited to no more than 10 (*K*_*a*_ ≤ 10).

The following shows some examples of *K*_*a*_ determination. Scenario A, B, and C are examples related to the America CPSIA and Canada CCPSA regulation, European Union REACH Directive Entry 51and52, and China standards and regulations GB 6675.1: 2014, respectively.

#### 2.3.1 Scenario A

Description: A DEHP is regulated at 0.1% with *U*_rel_ = 14%, *Q*_*M*_ = 2.4 mg/kg, *F* = 0.8, and the qualification rate *q* of the test portions in the batch is 99%.

*K*_*a*_ is determined as the following steps:

Step 1 *q* = 99%, according to Formula (2), *K*_*opt*_ = 11.

Step 2 *L* = 0.1%*, I* = 1*, U*_*rel*_ = 14%*, Q*_*M*_ = 2.4 mg/kg and *F* = 0.8, according to Formula (3), *K*_max_ = 286.

Step 3 *K*_*a*_ = Min (*K*_max_, *K*_*opt*_), which is 11. But due to *K*_*a*_ ≤ 10, the final composite group size *K*_*a*_ = 10.

#### 2.3.2 Scenario B

Description: Sum of DIBP, DBP, BBP and DEHP is regulated at 0.1% with *U*_*rel*_ of DIBP, DBP, BBP and DEHP is 13%, 15%, 18%, 14%, respectively. The *Q*_*M*_ of DIBP, DBP, BBP and DEHP is 2.5 mg/kg, 3.4 mg/kg, 20 mg/kg, 2.4 mg/kg and *F* = 0.8, the qualification rate, *q,* of the test portions in the batch is 95%.

Step 1 *q* = 95%, according to Formula (2), *K*_*opt*_ = 5.

Step 2 *L* = 0.1%*, I* = 4*, U*_*rel max*_ = 18%, *Q*_*M,max*_ = 20 mg/kg and *F* = 0.8, according to Formula (3), *K*_max_ = 8.

Step 3 The final composite group size *K*_*a*_ = Min (*K*_max_, *K*_*opt*_), which is 5.

#### 2.3.3 Scenario C

Description: Sum of DNOP, DINP and DIDP is regulated at 0.1% with *U*_*rel*_ of DNOP, DINP and DIDP is 21%, 23%, 23% respectively. The *Q*_*M*_ of DNOP, DINP and DIDP is 9.5 mg/kg, 41 mg/kg, 65 mg/kg and *F* = 0.8, the qualification rate *q,* of the test portions in the batch is 90%.

The following is the steps to determine *K*_*a*_:

Step 1 *q* = 90%, according to Formula (2), *K*_*opt*_ = 4.

Step 2 *L* = 0.1%*, I* = 3*, U*_*rel max*_ = 23% and *Q*_*M,max*_ = 65 mg/kg, and *F* = 0.8, according to Formula (3), *K*_max_ = “-” is obtained, and indicates that the composite test is not applicable.

Step 3 Individual tests need to be conducted in this case.

### 2.4 Calculation and judgment of testing results

As quantitative composite testing cannot provide the concentration of the target substance in each individual sub-sample, it is assumed that the detected target substance originates entirely from the sub-sample with the minimum mass when interpreting the detection results. To determine the maximum possible concentration, *W*_max_ (mg/kg) of the target substance that may exist in a single sub-sample, Formula (7) can be utilized. By comparing *W*_max_ with the regulated limit, the testing results can be evaluated, and the presence of unqualified samples in the group can be determined.

$$W_{\max}=c\times \frac{V}{m_{\min}}\times D$$ (7)

In this formula, *c* is the concentration of target substance in the solution to be tested following pretreatment of the sample group (mg/L); *V* is the constant-volume of the extraction liquid (mL); *D* is the dilution ratio; *m*_min_ is the minimum mass of a single sub-sample in the group.

Due to the uncertainty of each step in composite testing, it is necessary to correct the regulated limit (*L*) according to the measurement uncertainty to ensure the accuracy of the testing results. The calculation of the corrected limit, *L*_cor_, is given by Formula (8).

$$L_{cor}=L\times \left(1-U_{rel}\right)/I$$ (8)

If *W*_max_ ≤ *L*_cor_, all the samples in the group are qualified. Otherwise, if *W*_max_ > *L*_cor_, individual testing is required.

It is worth noting that in quantitative composite testing, if a composite sample group contains several low-concentration samples and each of these samples is below the corrected limit *L*_*cor*_, the maximum possible concentration *W*_max_ may be higher than *L*_*cor*_. This can result in a situation where all sub-samples in the group are qualified, but individual testing is still required to confirm whether there are unqualified samples. Therefore, it is not recommended to use quantitative composite testing when a large proportion of samples with low concentrations (50 or 100 mg/kg) are present. This situation should be identified through detection experiments of different detection items and is not included in this model.

### 2.5 Process of quantitative composite testing

**FIGURE 3:**
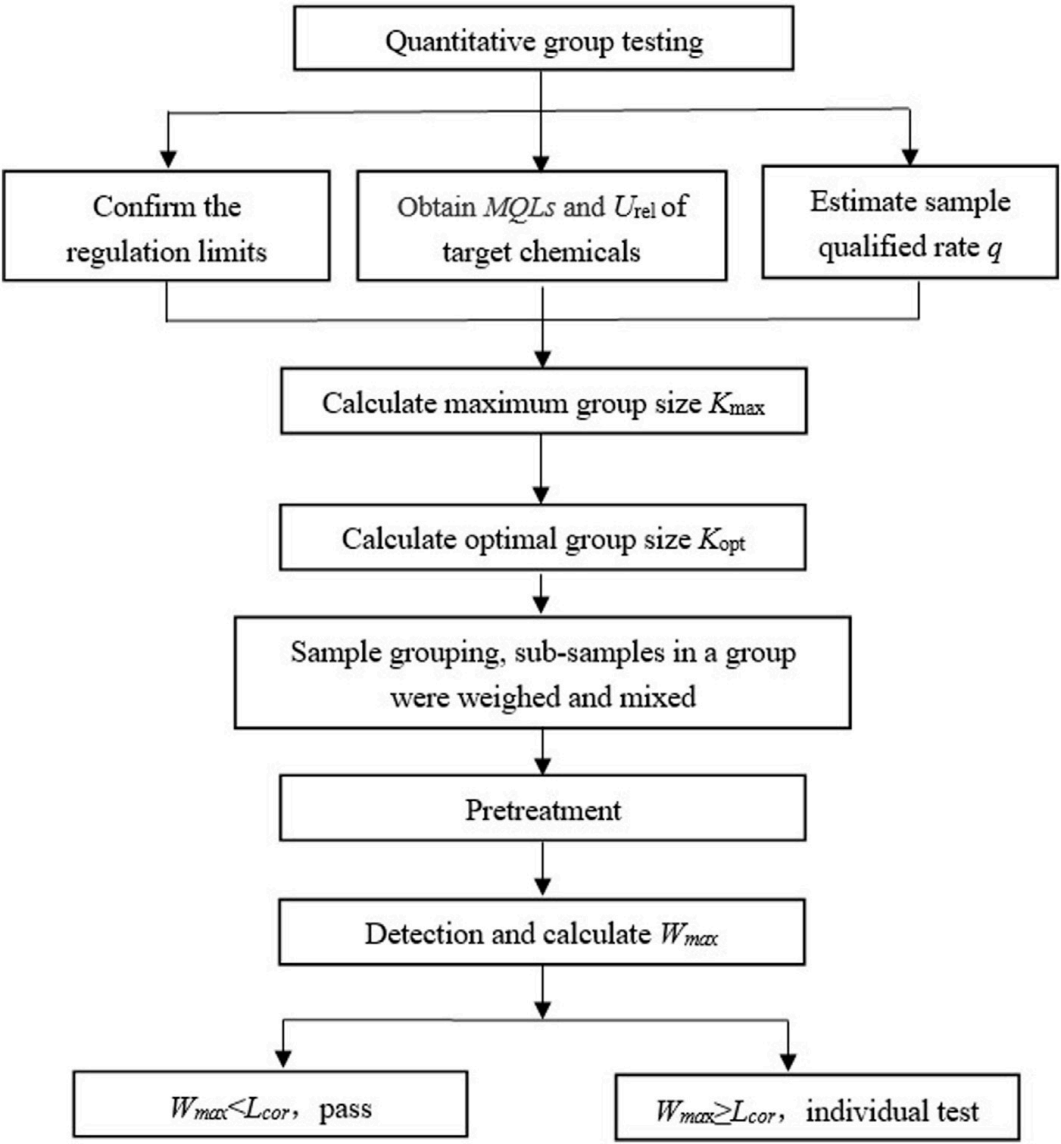
Flow chart to show the general quantitative composite testing with the constructed mathematical model.

Based on the constructed mathematical quantitative composite testing model, a flowchart of the work involved is presented in Figure 3. Firstly, the regulated limit *L*, *LOQ*, relative expanded measurement uncertainty *U*_*rel*_, and sample qualification rate *q* of the batch of samples need to be determined. With these parameters, *K*_max_ is calculated according to the model, and it is used to decide whether the quantitative composite testing can be conducted. If the maximum group size *K*_max_ ≤ 1, the method is not recommended. The optimal group size *K*_*opt*_ can be obtained from Table 1. The appropriate group size *K*_*a*_ can be selected as *K*_*a*_ = Min (*K*_max_ *, K*_*opt*_ *)* to enhance detection efficiency and ensure test accuracy. Next, *K*_*a*_ sub-samples are grouped, weighed, pretreated, and tested. Finally, the sample group’s qualification is determined by comparing the maximum possible concentration *W*_max_ with the corrected limit *L*_*cor*_. If the group may contain unqualified sub-samples, they are further tested individually.

## 3 Experimental validation

### 3.1 Instruments, materials and Reagents

The samples were weighed using a BS124S analytical balance (Germany Sartorius Group). PAEs were extracted from the samples using Elmasonic P type ultrasonic cleaner (Elma, Germany). The analysis of the selected PAEs was performed on a 7890A gas chromatograph hyphenated to a 5975C mass selective detector (Agilent Technologies, Palo Alto, CA).

7 PAEs standard products were purchased from Germany Dr. Ehrenstorfer Company, including Di-iso-butyl phthalate (DIBP, 99.39%), Di-n-butyl phthalate (DBP, 98.78%), Benzyl butyl phthalate (BBP, 99.39%), Di 2-Ethyl Hexyl Phthalate (DEHP, 99.39%), Di-n-octyl phthalate (DNOP, 99.39%), Di-iso-decyl phthalate (DIDP, 99.00%) and Di-iso-nonyl phthalate (DINP, 99.00%). Dichloromethane reagent (Chromatographically pure) was purchased from Fisher Scientific (United States of America).

There are 12 polyvinyl chloride (PVC)-base matrix samples, which include 9 blank ones (#1 ∼ #9) without PAEs and 3 positive samples (#A ∼ #C). #A is national certificated reference material (CRM) GSB 16-3484-2018; #B is business certificated material RMC (reference materials certificate) 010a; #C is quality control (QC) sample from the Technology Center of Guangzhou Customs District. The concentration of the 7 PAEs in the three positive samples are listed in Table 2.

**TABLE 2:** Reference value of 7 PAEs in the three positive samples (mg/kg).

No.	DIBP	DBP	BBP	DEHP	DNOP	DINP	DIDP
#A^[a]^	1200	2300	2050	1460	1800	1520	1160
#B^[b]^	926	1109	1076	980	1061	1041	1166
#C^[c]^	407	554	508	402	712	567	529

a These values of #A are from certificate of GSB, 16-3484-2018.

b These values of #B are from the business certificate of RMC, 010a.

c These values of #C are the average results of multiple parallel tests.

### 3.2 Preparation of Solution

20 mg of the 7 PAEs are respectively transferred into a 100 mL volumetric flask, by adding dichloromethane to the constant-volume line. Then, the PAEs were mixed stock standard solutions with a concentration of 200 mg/L. The standard working solutions are prepared as follows: firstly, transfer 10 mL of the stock standard solution into a 50 mL volumetric flask and add dichloromethane till the constant-volume line, until the solution reaches a concentration of 40 mg/L. Afterwards, transfer 1 mL, 2.5 mL, 12.5 mL, and 25 mL of these solutions into 100 mL volumetric flasks and add dichloromethane until the solution reaches the constant-volume line. Finally, the standard working solutions with concentrations of 0.4 mg/L, 1.0 mg/L, 5.0 mg/L, and 10 mg/L are obtained.

### 3.3 Sample pretreatment

The samples are cut into pieces with a diameter less than 5 mm. For each group, all the sub-samples should be weighed to 0.1 g (with a deviation within 10%) and mixed in a scintillation vial. Dichloromethane should then be added according to the total sample mass (i.e., 25 mL of dichloromethane should be added per 1 g of sample). The mixture should be subjected to ultrasonication in a water bath at 60 °C for 60 min. After the solution has cooled down, filter the supernatant through a 0.45 μm filter membrane.

### 3.4 GC-MS conditions

**FIGURE 4:**
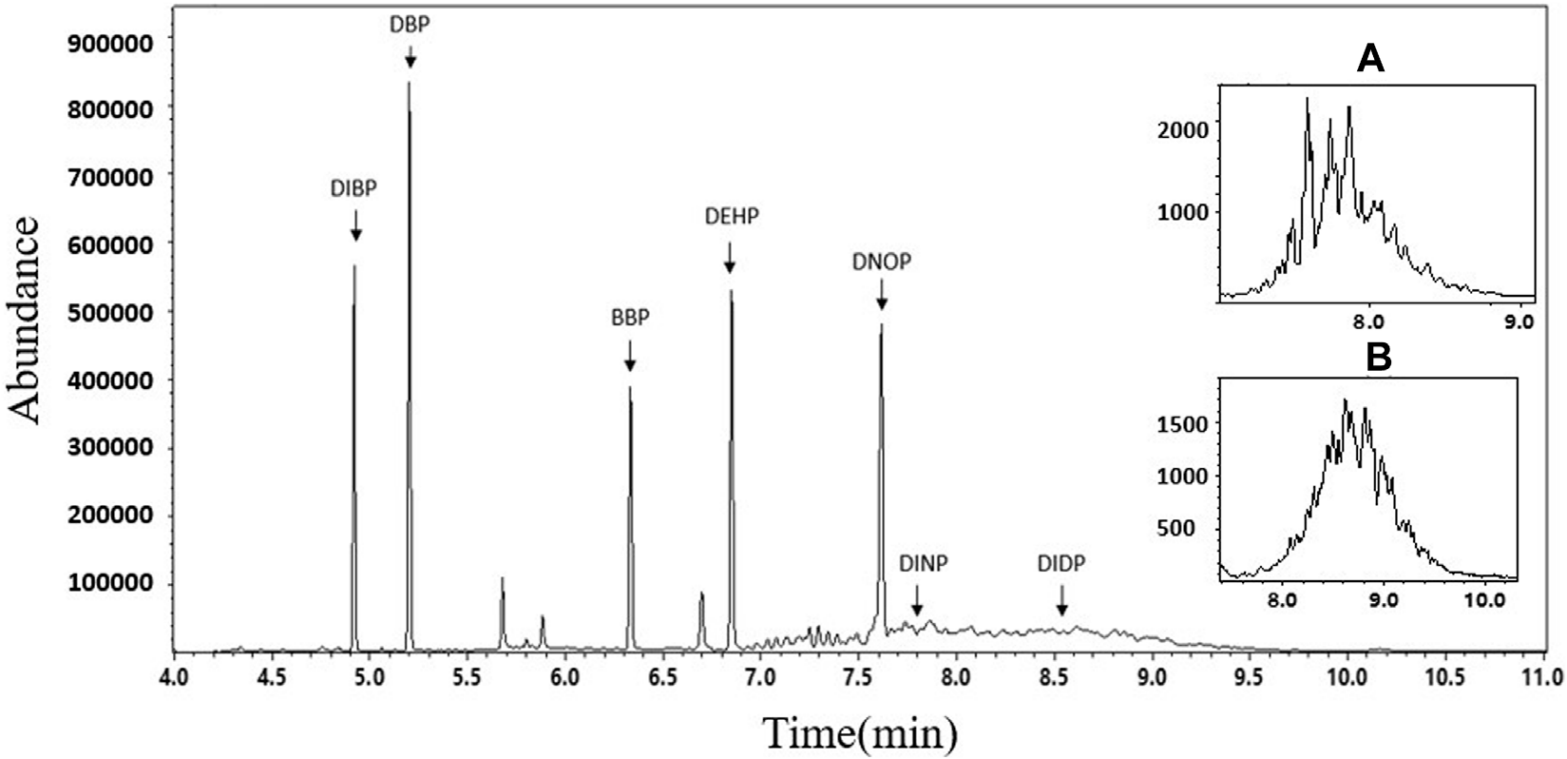
Total ion chromatogram of 7 PAEs. Detailed characteristic ion chromatograms of **(A)** DINP (m/z = 293) and **(B)** DIDP (m/z = 307).

The experimental conditions for gas chromatography-mass spectrometry (GC-MS) were based on the international standard ISO 8124-6. The GC-MS parameters and total ion flow chromatograms for the seven phthalate esters (PAEs) are presented in Table 3 and Figure 4, respectively. The GC separation of the PAEs was performed using a DB-5MS capillary column (30 m × 0.25 mm inner diameter × 0.25 μm film thickness) from Agilent J&W. Helium (99.999%) was used as the carrier gas and operated at a constant flow rate of 1 mL/min. The injector was operated in splitless mode at a temperature of 280°C, and the injection volume was set to 1 μL. The oven temperature program started at 80°C and ramped linearly to 290°C at a rate of 30°C/min and held for 1 min. The temperature was then increased to 300°C at a rate of 5°C/min and held for 3 min. The MS conditions included an ion source temperature of 280°C, an electron impact ionization source at 70 eV, and full scan mode ranging from m/z 50 to 500 were simultaneously applied for chemical determination using selected ion monitoring (SIM) mode.

**TABLE 3:** Retention time and characteristic ions of 7 PAEs.

No.	PAEs	Retention time (min)	Quantitative ion (m/z)	References ion
				(m/z)
1	DIBP	4.9	149	150,205
2	DBP	5.3	149	150,205
3	BBP	6.3	149	91,206
4	DEHP	6.8	149	167,279
5	DNOP	7.6	279	149
6	DINP	7.2 to 8.7	293	149
7	DIDP	7.5 to 9.6	307	149

## 4 Results and discussion

### 4.1 PAEs limit regulations

Currently, many countries have implemented strict regulations regarding the amount of PAEs allowed in toys and children’s products. Table 4 provides a list of some of the standard regulations and limit requirements of PAEs in consumer products including toys and children’s products issued by some countries and regions. The European REACH Directive is among the most stringent, mandating that the combined content of four PAEs (DEHP, BBP, DBP, and DIBP) in all toys and children’s products must not exceed 0.1%, while the combined content of three other PAEs (DINP, DIDP, and DNOP) in toys and children’s products that can be placed in the mouth must also not exceed 0.1% (European Agency for Safety and Health at Work, 2006; consumerfed, 2008; ASTM F963-2011, 2011; SOR/2016-188, 2017).

**TABLE 4:** Standards and corresponding regulations of PAEs in consumer products including toys and children’s products in various countries and regions.

Country/Region	Standards/Regulations	Limit
European Union	REACH Directive (Matsumoto et al., 2008)	1) DEHP + BBP + DBP + DIBP≤0.1%
		2) DINP + DIDP + DNOP≤0.1%
China	GB 6675.1-2014	1) DEHP + BBP + DBP≤0.1%
	GB 24613-2009	2) DINP + DIDP + DNOP≤0.1%
American	Consumer Product Safety Improvement Act (CPSIA) (consumerfed, 2008)	DIBP、BBP、DBP、DEHP、DCHP、DHEXP、DINP、DPENP≤0.1%
	ASTM F963-17 (ASTM F963-2011, 2011)	DEHP≤3%
Canada	SOR/2016-188 (Dodd et al., 2002)	1) DEHP,DBP,BBP≤0.1%
		2) DINP、DIDP、DNOP≤0.1%
Australia	Consumer Protection Act 2011	DEHP≤1%

### 4.2 Standard curve and method quantification limit

According to the constant volume used in *Sample Pretreatment (Section 3.3 of this study)* and regulatory limit requirements, the concentration range of the standard solution for the 7 PAEs was selected as 0.4–40 mg/L. The mixed standard solutions of 0.4 mg/L, 1.0 mg/L, 5.0 mg/L, 10.0 mg/L, and 40 mg/L, prepared in the *Preparation of Solution (Section 3.2 of this study)*, were quantitatively analyzed according to the instrument conditions specified in *GC-MS conditions (Section 3.4 of this study)*. Table 3 and Figure 4 present the GC parameters and total ion flow chromatograms of the 7 PAEs. The standard working curves of the 7 PAEs showed good linear relationships within the linear concentration range of 0.4–40 mg/L, and the linear correlation coefficients ranged from 0.9997-0.9999.

In composite testing, the mixing of multiple samples can lead to the dilution of the target object, which requires higher detection sensitivity compared to traditional single sample testing. Therefore, it is necessary to evaluate the *LOQ* in advance to determine whether it meets the requirements of group testing. To determine the *LOQ*, 10 μg of each of the 7 PAE standard substances was added to 1.0 g of a PVC blank sample (#1), and pretreatment was conducted according to *Sample Pretreatment (Section 3.3 of this study)*. The extracted liquid was then measured by GC-MS 7 times in parallel. The *LOQ* was determined as the 10 times standard deviation of the testing result of the target substance. The *LOQs* of the 7 PAEs were found to be 9.1–41.8 mg/kg, which were much lower than the limit requirements of 1000 mg/kg for the summation of 1–4 of PAEs in the China national standards and regulations given in Table 4. Therefore, they easily meet the needs of general detection.

### 4.3 Measurement uncertainty of composite testing

The calculated relevant uncertainty components: *u*_rel(m)_, *u*_rel(V)_, *u*_rel(std)_, *u*_rel(rec)_, *u*_*rel*(rsd)_ and the relative expanded uncertainty *U*_rel_ are presented in Table 5. Detailed calculation methods are presented in *The Maximum Allowable Group Size K*_*ma*x_ *(Section 2.2 in this study).* The relative expanded uncertainties *U*_rel_ of the 7 PAEs are 16.6%–23.2%. Then, the maximum allowable group size, *K*_max_, can be calculated based on the regulated limit *L*, Limit of quantification *LOQ,* and relative expanded uncertainty *U*_rel_ using Formula (3).

**TABLE 5:** The relevant uncertainty components u_rel_ and the relative expanded uncertainty *U*_rel_ of 7 PAEs (%).

PAEs	*u*_rel(m)_	*u*_rel(V)_	*u*_rel(std)_	*u*_rel(rec)_	*u*_rel(rsd)_	*u*_rel_	*U*_rel_
DIBP	0.17	1.2	5.3	3.1	7.6	9.8	19.7
DBP	0.17	1.2	5.3	2.3	7.3	9.4	18.8
BBP	0.17	1.2	5.3	2.7	6.8	9.1	18.2
DEHP	0.17	1.2	5.3	2.8	7.1	9.4	18.7
DNOP	0.17	1.2	5.3	2.3	5.8	8.3	16.6
DIDP	0.17	1.2	5.3	3.0	9.1	11.0	22.0
DINP	0.17	1.2	5.3	3.6	9.6	11.6	23.2

### 4.4 Calculation and judgement of composite testing results

Based on the limit requirements of the content summation of 1–4 PAEs in toys and children’s products in Table 4, the total amount of these PAEs should not exceed 0.1% (1000 mg/kg). In the quantitative composite testing method for the 7 PAEs, the maximum *LOQ* (*Q*_*M,max*_) is 41.8 mg/kg and the maximum relative expanded uncertainty (*U*_*rel*_) is 23.2%. Using Formula (3), the maximum allowable group size (*K*_max_) is calculated to be 4 or 18 (corresponding to *I* values of 1 or 4), respectively. Since the detection sensitivity meets the requirements of composite testing (*K*_max_ ≥ 2), this method can accurately determine whether composite samples contain any unqualified samples (i.e., samples in which one or more PAEs exceeds the limit).

### 4.5 Method accuracy

To verify the accuracy of the quantitative composite testing method for PAEs in toys, testing results were obtained using different group sizes *K*. A total of 12 PVC samples were used for composite testing, with detailed information given in *Instruments, Materials, and Reagents (Section 3.1 in this study)*. Sample grouping is listed in Table 6, with 6 sample groups of G1∼G6 set up with group sizes *K* of 3, 6, and 10, respectively. For each group size, there were 2 parallel groups. After pretreatment, GC-MS detection was performed, and the testing results are shown in Table 7.

**TABLE 6:** Grouping of PVC samples.

No.	Group size (*K*)	#A	#B	#C	#1	#2	#3	#4	#5	#6	#7	#8	#9
G₋1	3	₋^a^	**+** ^a^	**₋**	**+**	**+**	**₋**	**₋**	**₋**	**₋**	**₋**	**₋**	**₋**
G₋2	3	**₋**	**₋**	**+**	**+**	**+**	**₋**	**₋**	**₋**	**₋**	**₋**	**₋**	**₋**
G₋3	6	**+**	**₋**	**₋**	**+**	**+**	**+**	**+**	**+**	**₋**	**₋**	**₋**	**₋**
G₋4	6	**+**	**₋**	**+**	**+**	**+**	**+**	**+**	**₋**	**₋**	**₋**	**₋**	**₋**
G₋5	10	**₋**	**+**	**₋**	**+**	**+**	**+**	**+**	**+**	**+**	**+**	**+**	**+**
G₋6	10	**₋**	**+**	**+**	**+**	**+**	**+**	**+**	**+**	**+**	**+**	**+**	**₋**

^a^ “**+**” and “**-**” mean the group contains or does not contain the #No sample, respectively.

**TABLE 7:** The quantitative composite testing results *W*_max_ (mg/kg) of group samples.

No.	K	Sample composition	PAEs	DIBP	DBP	BBP	DEHP	DNOP	DINP	DIDP
G-1	3	0.1054g #B+ 0.1969g blank sample	References value	926	1109	1076	980	1061	1041	1166
			Measured value	1017	1025	1129	987	1143	1169	1061
			Recovery rate	109.8%	92.4%	104.9%	100.7%	107.7%	112.3%	91.0%
G-2	3	0.0950g #C + 0.2065g blank sample	References value	407	554	508	402	712	567	529
			Measured value	440	565	557	394	732	561	530
			Recovery rate	108.1%	102.0%	109.6%	98.0%	102.8%	98.9%	100.2%
G-3	6	0.1052g #A+ 0.5060g blank sample	References value	1200	2300	2050	1460	1800	1520	1160
			Measured value	1243	2362	2085	1470	1829	1491	1070
			Recovery rate	103.6%	102.7%	101.7%	100.7%	101.6%	98.1%	92.2%
G-4	6	0.1028g #A+ 0.1018g #C + 0.4062g blank sample	References value	1619	2877	2578	1877	2529	2102	1700
			Measured value	1603	2822	2506	1817	2694	2048	1660
			Recovery rate	99.0%	98.1%	97.2%	96.8%	106.5%	97.4%	97.6%
G-5	10	0.0986g #B+ 0.9220g blank sample	References value	926	1109	1076	980	1061	1041	1166
			Measured value	1008	1161	1045	1026	1078	1135	1148
			Recovery rate	108.9%	104.7%	97.1%	104.7%	101.6%	109.0%	98.5%
G-6	10	0.1013g #B+ 0.1027g #C + 0.7994g blank sample	References value	1338	1671	1591	1388	1783	1616	1702
			Measured value	1435	1689	1534	1394	1795	1662	1721
			Recovery rate	107.2%	101.1%	96.4%	100.4%	100.7%	102.8%	101.1%

In Table 7, the reference value of the group was calculated based on the reference PAEs value of the positive sample in Table 2. Using the concentration of each of the 7 PAEs, their corresponding measured value was calculated according to Formula (4). According to Table 7, the quantitative composite testing recovery rates are 91.0–112.3%, and the relative deviations between the measured values and their corresponding reference values are no more than 10%. Additionally, there were no false-positive and false-negative detection results. The variation of the group size had no significant effect on the testing result, indicating the accuracy of the PAEs quantitative composite testing method.

### 4.6 Computational verification

To investigate the false-negative and false-positive cases in the composite testing with a large number of samples, this study analyzed the PAE content in approximately 130,000 toys and children’s products from the baby product lab of the Technology Center of Guangzhou Customs District. Based on the practical statistical results, a simulated database with millions of samples was constructed and the random sample computer simulated group tests according the composite test model were conducted to verify the effectiveness and accuracy of the test results. The results indicate that both the false-positive and false-negative rates compared with the individual tests are very low and are within a controllable range. We have reported it in the ISO/TC 181 (Toy safety technical committee)/WG6 (Toy phthalates working group) meeting in 2021 and we will publish it in another paper. More detailed findings from this study will be reported elsewhere.

## 5 Conclusion

In order to improve the detection efficiency, a mathematical model of quantitative composite testing has been constructed based on measurement uncertainty. This model provides the applicable scope of composite testing, as well as the optimal number of composite samples for the sample group, and the calculation and judgment method of testing results. This composite testing model is a reference for the application of quantitative group testing methods in the field of quantitative analysis and detection of chemical substances. Furthermore, the mathematical model was applied to the PAEs composite testing of toy materials, and the experimental results showed that the *LOQ*s of 7 PAEs ranged from 9.1 to 41.8 mg/kg, which were much lower than the limits required in relevant standards and regulations. The relative expanded uncertainties were 16.6%–23.2%. Based on the mathematical model and the above parameters, the detection system sensitivity of the PAEs testing method met the requirements of quantitative composite testing. The recovery rates for PAEs quantitative composite testing with the group size *K* from 3 to 10 were 91.0%–112.3%, and the relative deviations were less than 10%, confirming the accuracy of the testing results.

When the *LOQ* is far lower than the regulation limit and the sample qualification rate is high, quantitative composite testing has extremely high application value, and it can greatly improve the detection efficiency and reduce the testing costs compared with traditional individual sample testing. The constructed model can be used not only in the quantitative testing of PAEs in toys, but also has the potential to be applied to the testing of PAEs in other materials. By adjusting the testing conditions, it can be used for other chemical substances, or even expand to quantitative composite testing of food, consumer goods, environment, and other fields.

The results of this research provide effective support for the establishment of a revised standard for the quantitative composite testing method about toys and children’s products. The revised standards will improve the quantitative detection method which breaks through the mixture sample limit of three, and the maximum allowable number of composite samples has been increased to 10. The theoretical calculation method and quick reference table for selecting the number of composite samples according to different parameters in quantitative testing were established for the first time and can effectively improve detection efficiency.

## Data Availability

The original contributions presented in the study are included in the article/supplementary material, further inquiries can be directed to the corresponding authors.
